# Infantile Hepatitis B in Immunized Children: Risk for Fulminant Hepatitis and Long-Term Outcomes

**DOI:** 10.1371/journal.pone.0111825

**Published:** 2014-11-07

**Authors:** Yu-Ru Tseng, Jia-Feng Wu, Man-Shan Kong, Fu-Chang Hu, Yao-Jong Yang, Chun-Yan Yeung, Fu-Chen Huang, I-Fei Huang, Yen-Hsuan Ni, Hong-Yuan Hsu, Mei-Hwei Chang, Huey-Ling Chen

**Affiliations:** 1 Department of Pediatrics, National Taiwan University Hospital, Taipei, Taiwan; 2 Department of Pediatrics, Chang-Gung Memorial Hospital, Taoyuan, Taiwan; 3 Department of Medical Research, National Taiwan University Hospital, Taipei, Taiwan; 4 Department of Pediatrics, Institute of Clinical Medicine, College of Medicine, National Cheng-Kung University and Hospital, Tainan, Taiwan; 5 Department of Pediatrics, Mackay Memorial Hospital, Taipei, Taiwan; 6 Department of Pediatrics, Chang-Gung Memorial Hospital-Kaohsiung Medical Center, Chang Gung University College of Medicine, Kaohsiung, Taiwan; 7 Department of Pediatrics, Veterans General Hospital, Kaohsiung, Taiwan; 8 Department of Medical Education and Bioethics, National Taiwan University, College of Medicine, Taipei, Taiwan; 9 Hepatitis Research Center, National Taiwan University Hospital, Taipei, Taiwan; Singapore Institute for Clinical Sciences, Singapore

## Abstract

**Background:**

Infantile hepatitis B after neonatal immunoprophylaxis is a rare yet distinct disease. This study aimed to analyze the long-term outcomes and risk factors in immunized infants with hepatitis B.

**Methods:**

The clinical parameters and outcomes of 41 infants born after universal immunization, and admitted for HBV-positive hepatitis were studied. All patients were followed for at least 6 months (median  = 4.4 years, range 0.6–18.1 years). Patient survival, changes of HBsAg and HBeAg status, and complications were analyzed.

**Results:**

Among the 41 cases (32 males, 9 females), 21 presented with fulminant hepatitis (FH), and 20 with non-fulminant hepatitis (NFH). Ninety-five percent (36/38) of the mothers were positive for hepatitis B surface antigen (HBsAg). Multivariate analyses revealed younger age of onset (age <7 months) and negative maternal hepatitis B e antigen (HBeAg) were associated with FH (p = 0.03 and p = 0.01, respectively). An infantile fulminant hepatitis B risk score using maternal/infant HBeAg positivity and onset age was proposed. Among the FH cases, the rate of mortality, HBsAg clearance, and chronic HBV infection were 47.6%, 38.1%, and 14.3%, respectively. Among the NFH cases, 35% developed chronic infection. Of the 9 chronically infected children received long-term follow-up, 8 had HBeAg seroconversion before 4 years of age. One case of FH developed hepatocellular carcinoma 14 years later.

**Conclusions:**

Maternal HBsAg + /HBeAg- and early onset age were risk factors for FH in immunized infants. A significant portion of patients with FH or NFH evolve to chronic HBV infection, with HBeAg seroconversion in young childhood. Close surveillance for hepatocellular carcinoma is warranted in patients surviving infantile hepatitis B.

## Introduction

Hepatitis B virus (HBV) infection is a worldwide health problem. Approximately 350 million people are chronically infected, and 600,000 deaths are caused by HBV-related hepatic failure, cirrhosis, or hepatocellular carcinoma (HCC) annually [1]. Acute HBV infection may manifest asymptomatic infection, hepatitis, or fulminant hepatitis (FH), with a mortality rate of FH up to 80%. In the survivors of FH, most cases recover without the development of chronicity [2]–[4].

Universal HBV immunization has been shown to be highly effective in preventing mother-to-infant HBV transmission and to significantly reduce the HBV carrier rate in many countries [5]–[7]. In Taiwan, a mass Hepatitis B immunization program has been launched since 1984 [8]. The incidence of chronic HBV infection has been markedly decreased from 10% to below 1%, with a high vaccination coverage rate of 90∼98% [9], [10]. A decrease in the number of acute hepatitis B cases has been noted under immunization program [11], [12]. However, HBV immunization cannot eradicate HBV infection completely. We have reported that 9.26% of the children born to hepatitis B e antigen (HBeAg)-positive/hepatitis B surface antigen (HBsAg)-positive mothers became chronically infected with HBV [13]. Importantly, about one-third of HCC cases cannot be prevented [14], [15].

HBV infection in infancy is a unique condition. Most infected infants acquire HBV infection asymptomatically during perinatal period, have normal alanine aminotransferase (ALT) levels in the acute period and then progress to chronic infection [16]. However, infants born to HBeAg-negative, HBsAg-positive carrier mothers are at risk for the development of fulminant hepatitis B [17], [18]. In the post-immunization era, we have previously reported that HBV infection is still an important cause of infantile FH [19]. However, aside from our previous nationwide survey, only a few other studies have reported a total of less than 10 cases of infantile FH caused by HBV [20]–[22]. The clinical course and long-term outcomes of hepatitis B in infancy, especially among those patients after hepatitis B immunization, are unclear. We therefore conducted this study to delineate the risk factors and long-term outcome of fulminant and non-fulminant hepatitis B in infants in the era post universal immunization.

## Methods

### Subjects

A retrospective, hospital-based study was conducted from 6 tertiary medical centers in Taiwan. A total of 41 infants aged 1–12 months who had been admitted to the hospital between 1986 and 2006 due to hepatitis with an initial ALT level >100 U/L and a positive HBsAg or IgM antibody to hepatitis B core antigen (anti-HBc IgM) were included. Infants less than 1 month old were not included in this study to exclude the possibility of inborn errors of metabolism and perinatal insults. The clinical characteristics, serum biochemistry, HBV markers of children and mothers, and immunization histories of the children were recorded.

### Definition of fulminant hepatitis

FH was defined as the development of the following: 1). hepatic-based coagulopathy that was not corrected by parenteral administration of vitamin K; and 2). hepatic encephalopathy that was present if the uncorrected prothrombin time or international normalized ratio (INR) was between 15 and 19.9 seconds or 1.5 to 1.9, respectively. Hepatic encephalopathy was not required if the PT was greater than or equal to 20 seconds or the INR was greater than or equal to 2.0, within 8 weeks of disease onset according to the Pediatric Acute Liver Failure (PALF) Study Group [23]. Those patients who did not meet the FH definition criteria were classified as the non-fulminant hepatitis (NFH) group.

### Follow-up and outcomes

Cases in the FH group were defined as non-survivors if death occurred during the first hospitalization. The surviving cases in the FH group and all cases in the NFH group had a follow-up duration of more than 6 months. Recovery was defined as the loss of HBsAg positivity within 6 months. Chronic hepatitis B infection was defined as the seropositivity of HBsAg for more than 6 months. HBeAg seroconversion was defined as the loss of HBeAg positivity and appearance of antibody to HBeAg (anti-HBe). This study was approved by the National Taiwan University Hospital Research Ethics Committee (approval number: 200909049R), without written informed consent. Patient records/information was anonymized and de-identified prior to analysis.

### HBV immunization program and immunization status

The HBV immunization program in Taiwan was launched in July 1984 [8]–[10]. In brief, maternal HBsAg and HBeAg were tested before 32–34 weeks of gestation. Three doses of HBV vaccines were given at age 0, 1, and 6 months. For neonates of HBeAg-positive mothers, hepatitis B immunoglobulin (HBIG) was given within 24 hours of birth. The option of receiving self-paid HBIG for infants born to HBeAg-negative, HBsAg-carrier mothers is provided in most hospitals. Patients in this study were defined as been immunized on schedule if they received the appropriate immunization for their age as recommended by the government before the diagnosis of hepatitis.

### Statistical analysis

Data analysis was performed using SAS 9.1.3 (SAS Institute Inc., Cary, NC U.S.A.) and R 2.9.2 (Free Software Foundation, Inc., Boston, MA U.S.A.) software. Clinical variables, including age of onset, gender, immunization status, maternal HBV serostatus, and initial and peak blood biochemistry data (ALT, total bilirubin, INR) were compared between the FH and NFH group. These variables were also analyzed for their association with long-term outcomes in both the FH group and the NFH group.

To compare characteristics between groups, the chi-square test or Fisher's exact test was used to analyze categorical variables, and the Mann–Whitney two-sample rank-sum test or the Kruskal-Wallis test applied for continuous variables. Multivariate analyses using logistic regression models were applied to identify the following: (1) the risk factors associated with the occurrence of FH; (2) the predictors of prognosis (recovery, carrier, or death). When separation problem occurred, the exact logistic regression method was used. The generalized additive models (GAMs) were applied to detect nonlinear effects of continuous covariates, if necessary. To ensure the quality of the analysis results, basic model-fitting techniques for (1) variable selection, (2) goodness-of-fit (GOF) assessment, and (3) regression diagnostics were used in our logistic regression analyses. Specifically, the stepwise variable selection procedure (with iterations between the forward and backward steps) was applied to obtain the candidate final regression model. A *p* value less than 0.05 was considered statistically significant.

## Results

### Clinical characteristics in patients with FH and NFH

Among the 41 subjects, 21 infants presented with FH and 20 presented with NFH, with male predominance (78%, *P*<0.001). Clinical characteristics of the two groups are shown in Table 1. The median age at onset in the FH group was 3 months (range  = 1.2–6.4), which was younger than the age at onset in the NFH group (medium  = 5.6 months, range  = 2.0–11.7, *P* = 0.001). All these children were Taiwanese. Of the 19 patients with genotype tested, 15 were genotype B, 2 were genotype C, and 2 were mixed genotype B and C.

**Table 1:** Clinical characteristics of 41 infants with acute or fulminant hepatitis B.

	NFH group (n = 20)	FH group (n = 21)	*p*-value
**Demographic data**			
Sex (M/F)	15/5	17/4	0.71
Age of onset (m)	5.6 (2.0–11.7)	3 (1.2–6.4)	0.001
**Maternal HBV status**			
Maternal HBsAg (+)	16/18 (89%)	20/20 (100%)	0.218
Maternal HBeAg (+)	11/18 (61%)	2/20 (10%)	0.001
**Vaccination status**			
HBIG (+)	10/18 (56%)	2/20 (10%)	0.003
HBV vaccine			0.09
On-schedule	14/17 (82.3%)	20 (100%)	
Off-schedule	3/17 (17.6%)	0	
**Clinical data**			
HBeAg (+) at admission	10/17 (59%)	3/18 (17%)	0.01
Initial ALT (IU/L)	758(79–3020)	1688 (286–4191)	0.002
Initial total bilirubin (mg/dl)	3.4(0.2–11.5)	10.3 (4.4–15)	<0.001
Initial INR (s)	1.40 (0.9–1.9)	5.6 (1.1->10)	<0.001
Peak ALT level (IU/L)	821(129–3020)	1716(326–4289)	0.001
Peak total bilirubin (mg/dl)	4.2 (0.4–14.6)	24.8 (7.6–53.5)	<0.001
**Survival**	20	11	<0.001

FH: fulminant hepatitis, NFH: non-fulminant hepatitis, HBsAg, hepatitis B surface antigen; HBeAg, hepatitis B e antigen, HBV, hepatitis B virus; HBIG, hepatitis B immunoglobulin; ALT, alanine aminotransferase; INR, international normalized ratio.

#### Maternal HBV status

Ninety-five percent (36/38) of mothers with known HBV status were HBsAg carriers. Maternal HBeAg was positive in 10% (2/20) of FH cases, whereas it was positive in 61% (11/18) of NFH cases (*P* = 0.001, Table 1).

#### HBV immunization status

HBV immunization status was documented in 37 cases. In the FH group, all patients had received HBV immunization as scheduled. In the NFH group, immunization was delayed in 3 cases (17.6%, *P* = 0.09), with disease onset at 3 (received only 1 dose of HBV vaccine), 5 (delayed second dose at 2-months-old) and 7 months (received two doses only due to Kawasaki disease and administration of intravenous immunoglobulin), respectively. The HBIG immunization rate was higher in the NFH group (*P* = 0.003, Table 1), which was likely due to the higher maternal HBeAg-positive rates and the current immunization strategy in Taiwan to give HBIG only to infants born to mothers who are positive for both HBsAg and HBeAg.

#### Laboratory tests

Significant differences were found between groups in the serial laboratory test results (Table 1). The rate of patient HBeAg positivity in the index patients at first blood sample was 59% in the NFH group and 17% in the FH group (*P* = 0.01). Univariate analyses indicated that the initial and peak blood biochemistry results (ALT and total bilirubin) and coagulation profiles were significantly more severe in the FH group than in the NFH group.

### Predictors of fulminant hepatitis and risk score

In all 41 patients, multivariate analyses of the risk factors for the development of FH were applied by fitting logistic regression models using the stepwise variable selection method. All of the clinical variables in Table 1 except INR were considered in the models because INR was the diagnostic criterion for FH. Age of onset was found to be the separation factor in model 1 (OR = 1.6, *P* = 0.0385, Table S1) because the onset age in the FH group was universally less than 7 months. After limiting the analyses to those subjects whose onset age was less than 7 months, maternal HBeAg became the separation factor in model 2. Negative maternal HBeAg was associated with the development of FH (OR = 10.8, *P* = 0.0132).

Based on the results of the above analyses, we developed a scoring system to predict the development of FH, using three parameters: onset age less than 7 months, negative maternal HBeAg, and infant HBeAg status at the first blood sampling (Table 2 and 3). Good predicting capacities (AUC  = 0.875, Hosmer and Lemeshow Goodness-of-Fit test, *P* = 0.2632) were found. Using score  = 4 calculated from this “infant fulminant hepatitis B (IFHB) Risk Score” to predict FH, the sensitivity, specificity, positive predictive value, negative predictive value, and diagnostic accuracy were 82.3%, 86.7%, 87.5%, 81.2% and 84.4%, respectively.

**Table 2:** The infant fulminant hepatitis B (IFHB) Risk Score to predict fulminant hepatitis B in infancy.

Parameters	Score
Negative maternal HBeAg*	1
Age <7 month-old#	2
Negative HBeAg in the patient$	1

*The score was 0 for positive maternal HBeAg patients and 1 for negative maternal HBeAg patients.

# The score was 0 for onset age ≥7 month-old and 2 for onset age <7 month-old.

$ The score was 0 for positive HBeAg patients and 1 for negative HBeAg patients.

**Table 3:** The IFHB Risk Scores in the infantile cases of hepatitis B.

Score	FH group (N = 17)	NFH group (N = 15)
0	1	6
1	0	1
2	0	4
3	2	1
4	14	3

FH, fulminant hepatitis; NFH, non-fulminant hepatitis.

### Survivals, outcomes, and predicting factors

The mortality rate of the FH patients was 47.6% (10/21). All the mortality occurred within 6 months after disease onset. No patient had received liver transplantation. The median follow-up duration of the surviving patients (11 FH and 20 NFH patients) was 5.0 years (0.6–18.1 years).

**Figure 1:**
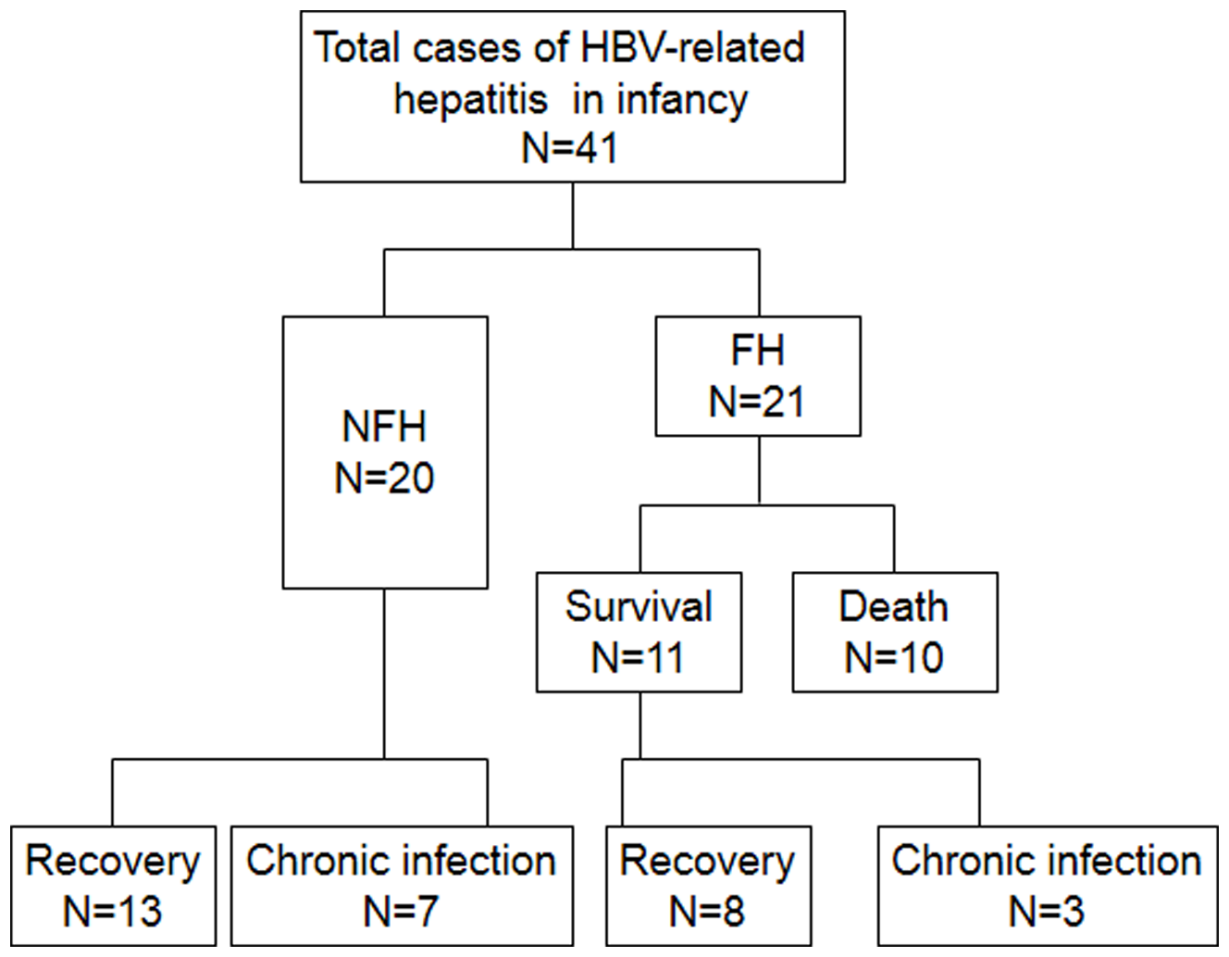
Clinical course and outcomes of infants with hepatitis B; FH, fulminant hepatitis; NFH, non-fulminant hepatitis. Recovery is defined as HBsAg seroclearance. Chronic infection is defined as persistence of HBsAg for more than 6 months.

In the FH group, 38.1% (8/21) had seroclearance of HBsAg, and 14.3% (3/21) became chronic HBV carriers (Figure 1). In univariate analyses, age of enrollment, HBeAg at onset, initial INR, peak INR, and peak total bilirubin level played as important factors of the development of chronic HBV carrier and mortality (Table 4). Multivariate logistic regression analyses revealed that high initial INR was associated with the development of chronic HBV infection and mortality (OR: 1.31, *P* = 0.0424) (Table S2).

**Table 4:** Clinical characteristics and outcome of 21 infants with fulminant hepatitis B.

	Recovery (n = 8)	Chronic carrier (n = 3)	Mortality (n = 10)	*P*-value
**Demographic data**				
Sex (M/F)	5/3	3/0	9/1	0.308
Age of onset (m)	2.93 (1.72–4.77)	6.28 (5.49–6.44)	2.8 (1.2–3.9)	0.02
**Maternal HBV status**				
Maternal HBsAg (+)	8/8 (100%)	2/2 (100%)	10/10 (100%)	1
Maternal HBeAg (+)	0/8 (0)	1/2 (50%)	1/10 (10%)	0.19
**Clinical data**				
HBeAg (+) at enrollment	1/7 (14%)	2/3 (67%)	0/8 (0)	0.02
Initial ALT level (IU/L)	1657 (286–4191)	1628(1508–1720)	1716 (969–2923)	0.84
Initial total bilirubin (mg/dl)	8.6 (4.6–21.6)	5.6 (5.3–29.5)	10.1(1.3–15)	0.44
Initial INR	2.5 (1.16–8.35)	3.87 (1.13–10)	9.0 (2.3->10)	0.06
Peak INR	3.5 (1.16–12.5)	4.08 (1.13–10)	9 (2.6->10)	0.06
Peak ALT level (IU/L)	1945 (326–4289)	1720 (1508–2465)	1716 (1026–2923)	0.98
Peak total bilirubin (mg/dl)	16.8 (7.6–36.2)	16.2 (10.4–32.4)	39 (22–53.5)	0.01

HBsAg, hepatitis B surface antigen; HBeAg, hepatitis B e antigen, HBV, hepatitis B virus;

HBIG, hepatitis B immunoglobulin; ALT, alanine aminotransferase; INR, international normalized ratio.

In the NFH group, 13 cases (65%) recovered from hepatitis B, while 7 cases (35%) had chronic HBV infection (Figure 1). All the chronic HBV carriers had HBeAg-positive mothers, while 4 cases (36%) in the recovery group had HBeAg-positive mothers (*P* = 0.01). The initial HBeAg status of the patients was positive in all (7/7) the patients who became chronic carriers, and only 3/10 patients (30%) in the recovery group had initial HBeAg positivity (*P* = 0.01). Interestingly, higher levels of serum blood biochemistry markers were found in the recovery group, including initial/peak ALT, total bilirubin and INR (Table S3). Multivariate logistic regression analysis showed that a higher initial ALT level was associated with recovery state (*P* = 0.04).

Two patients in the FH group and two in the NFH group have received lamivudine treatment. One of the two FH patients survived and became HBsAg negative, the other died. Of the two NFH patients, one recovered and one became chronically infected.

**Figure 2:**
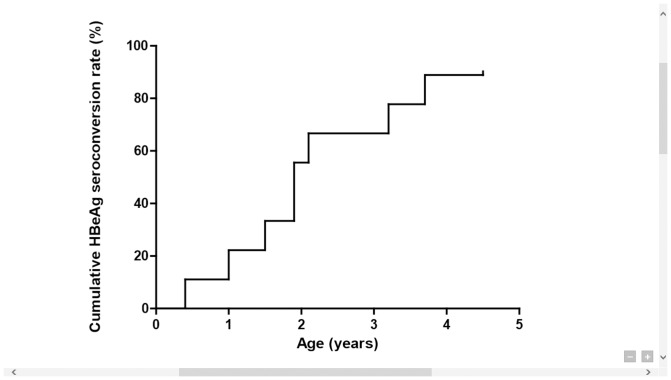
Cumulative HBeAg seroconversion rates in survivors of infantile fulminant/non-fulminant hepatitis B (N = 9).

A high rate of early HBeAg seroconversion was noted in the surviving patients of FH and NFH group who became chronic carriers. In the 10 subjects who progressed to chronic HBV infection, 9 had received long-term follow-up. Among these 9 cases, all had initial positive HBeAg except one unique case described below. Eight (89%) of them had HBeAg seroconversion before the age of 4 years (Figure 2).

### Clinical course of one fulminant hepatitis case with subsequent chronic HBV infection and HCC

**Figure 3:**
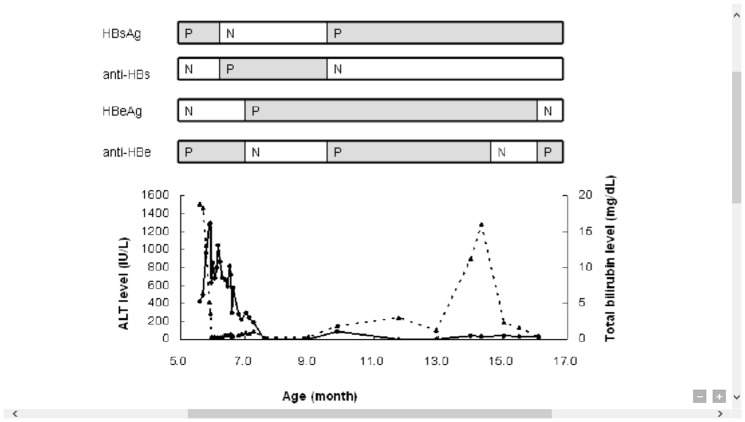
Serial data of a case of infantile fulminant hepatitis that became a chronic HBV carrier. HBsAg: hepatitis B surface antigen; anti-HBs: hepatitis B surface antibody, HBeAg: hepatitis B e antigen, anti-HBe: antibody to HBeAg, ALT: alanine aminotransferase level (dotted line), T-bil: total bilirubin(straight line); N: negative; P: positive.

This male infant was born to an HBeAg-negative, HBsAg positive mother and had received 2 doses of HBV vaccines on schedule before FH developed at the age of 5.6 months. Serum HBsAg and anti-HBe were positive, and HBeAg negative at the time of admission. HBsAg seroclearance and the development of anti-HBs were noted at the age of 6.6 months. However, HBeAg and HBsAg reappeared with elevation of aminotransferase levels at the age of 7–16 months, peak ALT of 1280 U/L. Mutations in the HBV precore region was tested for the first blood sample and a second sample when anti-HBe was negative. No precore mutation was detected in the two samples. We have also tested pre-S mutations and found T68I/L108I/A138G in the acute stage, and F141L in the convalescent stage. The mutation sites 108,138, and 141 were considered regions for B-cell epitope, and have not been found in previous reported cases of fulminant hepatitis [24], [25]. This infant achieved spontaneous HBeAg seroconversion at the age of 17 months (Figure 3). He received regular follow-up every 6 months, remained HBsAg positive/anti-HBe positive, normal ALT and alpha-fetoprotein levels for years. Elevation of alpha-fetoprotein (142 ng/ml) was noted during regular follow-up at the age of 14 years. HBV viral load was 618 IU/ml. Magnetic resonance image showed a foal mass lesion in the left medial and right anterior liver in T2WI image. Tumor resection was performed (a 4×3 cm tumor and a 1×1 cm satellite nodule). The pathology revealed trabecular HCC and mild fibrosis in the non-tumor liver. The alpha-fetoprotein was normal when followed 12 months after operation.

## Discussion

Current infant immunization program against HBV cannot completely eradicate fulminant hepatitis in infancy, which is a potentially fatal disease [19]–[22]. Previous data concerning the long-term outcomes of hepatitis B in infancy after immunization are lacking. It is generally considered that patients can produce HBsAg clearance and develop anti-HBs status, achieving virological recovery if surviving after severe hepatic inflammation. However, we found from this study that in those who had hepatitis B in infancy, 14.3% (3/21) of the patients with FH and 35% (7/20) of patients with NFH became chronically infected with HBV. Moreover, one patient developed HCC 14 years later, suggesting the long-term complication of hepatic inflammation in infancy.

In our and previous reports, the majority of fulminant hepatitis B in infancy were born to HBsAg positive/HBeAg negative mothers [17]–[19]. It is still debatable whether administration of HBIG at birth may have additional protection, under HBV vaccines coverage. HBIG administration has not totally prevented patients from developing fulminant hepatitis B [21], [22]. Estimations of cost-benefit of HBIG have been reported and indicated potential but controversial benefit of HBIG added on HBV vaccines in preventing fulminant hepatitis in children born to HBsAg positive/HBeAg negative mothers, with a cost-benefit ratio 1.36 (0–3.97 in the sensitivity analysis) [13]. Comparisons of cost-effectiveness of different immunization strategies have also been reported [26].

The development of chronic infection is reported to be less than 2.4% after acute hepatitis B, 3.3% after fulminant hepatitis in adult patients, and the chronicity rate may increase to 17% in immune-compromised patients [27], [28]. Children acquiring HBV in infancy caused by mother-to-infant transmission is a unique clinical condition, and is known to have highest rates of chronicity compared with those contracted infection at later [29]. The clinical presentation of infantile hepatitis in the current study ranged from 1.2 to 11.7 months of age, but the exact timing of transmission in these children could not be determined. It is therefore possible that these children may have intrauterine, perinatal, or postnatal infection. Mother-to-infant transmission of HBV mostly likely causes asymptomatic chronic infection, known as immune-tolerant phase with normal liver function in the infancy and childhood. Cases developing overt hepatitis are infrequent in infancy. However, the chance of developing fulminant hepatitis B is higher in infancy than in other age periods [19]. In particular, one patient in this study had HBsAg clearance and anti-HBs positivity 1 month after diagnosis of FH, but HBsAg reappeared 3 months later, indicating failure of host immune response to clear the virus. Viral replication 7 years after survival of fulminant hepatitis and the clearance of HBsAg has been reported in a case with infantile fulminant hepatitis B [30]. Interestingly, in the NFH group, lower ALT levels were associated with chronicity. It is speculated that host immunity might contribute to viral clearance and simultaneously produce modest liver damage. Milder elevation of transaminase levels may indicate a tendency to develop immune tolerance, as in most cases of chronic HBV infection.

The majority of our cases who developed chronic infection achieved HBeAg seroconversion before the age of 4 years, which is much earlier compared to maternally transmitted cases of chronic asymptomatic HBV infection, with a mean age of HBeAg seroconversion at 16.8±5.5 years [31]. A vigorous immune response during the course of hepatitis at a young age may have attributed to the very early HBeAg seroconversion seen in our study. HBeAg seroconversion has been considered a hallmark of the effectiveness of antiviral therapy for chronic HBV infection [32]. However, we have reported previously that HBeAg seroconversion in early childhood is associated with the early development of liver cirrhosis and HCC [33], [34]; the fact argues against the favorable outcome of very early HBeAg seroconversion.

We first report a unique case that developed HCC 14 years after survived from FH. This case evolved to chronic infection and had very early HBeAg seroconversion after infantile FH. Chronic HBV infection and its complication may often be ignored or missed in subjects with complete HBV immunization. In a previous nationwide survey, a reduction of incidence of HCC by two thirds, which was disproportionate to the 90% reduction of chronic HBV carrier rate in the population (from 10–20% to 1–2%) was found [10], [14], [15]. It is suggested that immunized children with breakthrough HBV infection may carry a higher risk of developing HCC than the HBsAg-carrier children born in the pre-immunization era [35]. The reason is speculated to be related to route of transmission. In the post-immunization era, horizontal infection is mostly blocked; and the majority (88.5%) of the chronically infected children is transmitted from their mothers [10]. Those with maternal HBsAg positivity have an odd's ratio of 29.5 to develop HCC compared with maternal HBsAg negative population [15]. Although most of the complications of chronic liver disease caused by HBV developed decades after contracting infection, the one adolescent patient that developed HCC in our report raise awareness of early onset liver complications in patients seemingly recovered from infantile hepatitis B. We emphasize the importance of surveillance of all children born to HBsAg carrier mothers [13], and as recommended by The US Advisory Committee on Immunization Practices (ACIP) to screen HBV infection for all children born to HBsAg mothers at age 9–18 months [36].

On the other hand, to optimize short-term survival, a timely referral of patients to a hospital with adequate intensive care facilities and evaluation for liver transplantation is important in the acute stage of infantile hepatitis B. Our IFHB risk score facilitates the identification of high risk patients for developing FH, using parameters that are easy to obtain at the time of admission, and are not fluctuated with the disease progression. Although maternal negative HBeAg and positive HBsAg has been described before in the pre-immunization era [18]–[20], the onset age (<7 months) has not been addressed in the infantile hepatitis caused by HBV. It has been reported that FH in infants born to HBeAg-negative mothers may be attributed to the lack of immunological tolerance induced by transplacental maternal HBeAg [37]. Infection of precore mutants strains has been reported in patients with fulminant hepatitis [20], [38], but subsequent studies had shown inconsistent results regarding the causative roles of precore mutants [39], [40]. Infection by pre-S2 defective virus, with host's immune hyper-response to the viral antigens has been proposed [24]. Whether negativity of HBeAg at diagnosis is the result of fast HBeAg seroconversion before diagnosis of FH due to vigorous immune responses, or the results of infection by pre-existing viral strain carrying mutations needs further investigation.

In conclusion, the present study showed that infants presenting with HBV infections post universal immunization program may run a fulminant, acute-resolving, or inflammation evolving to chronic infection course. A simple, rapid IFHB score for predicting the development of FH was developed, comprising negative maternal HBeAg, onset age <7 months, and negative patient HBeAg. Among the patients surviving infantile FH or NFH, a significant proportion developed chronic HBV infections and early HBeAg-seroconversion. We suggest routine screening of HBV markers for children born to HBsAg carrier mothers. Close follow-up and surveillance for chronic infection and HCC is especially warranted in patients seemingly recovered from with infantile hepatitis B.

## Supporting Information

Table S1 **Multivariate analysis of the predictors of fulminant hepatitis B by fitting logistic regression models using the stepwise variable selection method.**
(DOC)

Table S2 **Multivariate analysis of the predictors of prognosis in the fulminant hepatitis B group by fitting logistic regression models.**
(DOCX)

Table S3 **Clinical characteristics and outcome of 20 infants with non-fulminant hepatitis B.**
(DOCX)
