# Supplementary material for: Infantile Hepatitis B in Immunized Children: Risk for Fulminant Hepatitis and Long-Term Outcomes
Source: PLoS One. 2014 Nov 7;9(11):e111825. doi: 10.1371/journal.pone.0111825 (PMC4224399; doi:10.1371/journal.pone.0111825)
Supplement: Table S1 — Multivariate analysis of the predictors of fulminant hepatitis B by fitting logistic regression models using the stepwise variable selection method. (DOC) [file pone.0111825.s001.doc]

Table S1. Multivariate analysis of the predictors of fulminant hepatitis B by fitting logistic regression models using the stepwise variable selection method

| Covariate | Standard error | Wald  Chi-square | | p-value | Odds ratios | | 95% CI |
| --- | --- | --- | --- | --- | --- | --- | --- |
| **Model 1: Considering all available covariates, except INR** | | | | | | | |
| Age of onset (m)* ^#^ | 0.2397 | | 4.2838 | 0.0385 | 0.609 | 0.381- 0.974 | |
| **Model 2: Considering all available covariates except INR, and age of enrollment below 7 month-old** | | | | | | | |
| Maternal HBeAg^&^ | 0.9603 | | 6.1398 | 0.0132 | 0.093 | 0.014 -0.608 | |

* Age of onset was found to be separation factor in model 1, because all the onset age of the fulminant hepatitis group was under 7 month-old.

^#^ Multiple logistic regression model 1: *n* = 38, percentage of concordant pairs = 85.0%, percentage of discordant pairs = 14.7%, adjusted generalized *R*^2^ = 0.3830, Deviance goodness-of-fit test *p* = 0.5442 > 0.05 (df = 33), Pearson goodness-of-fit test *p* = 0.5165 > 0.05 (df = 33), and Hosmer and Lemeshow goodness-of-fit test *p* = 0.5453.05 (df = 7).

^&^Multiple logistic regression model 2: *n* = 34, percentage of concordant pairs = 49.1%, percentage of discordant pairs = 4.5%, percentage tied = 46.4, adjusted generalized *R*^2^ = 0.2083.
