# Supplementary material for: Infantile Hepatitis B in Immunized Children: Risk for Fulminant Hepatitis and Long-Term Outcomes
Source: PLoS One. 2014 Nov 7;9(11):e111825. doi: 10.1371/journal.pone.0111825 (PMC4224399; doi:10.1371/journal.pone.0111825)
Supplement: Table S2 — Multivariate analysis of the predictors of prognosis in the fulminant hepatitis B group by fitting logistic regression models. (DOCX) [file pone.0111825.s002.docx]

Table S2. Multivariate analysis of the predictors of prognosis in the fulminant hepatitis B group by fitting logistic regression models

| Covariate | Estimate | | Standard error | Wald  Chi-square | | p-value | | Odds ratios | | 95% CI | |
| --- | --- | --- | --- | --- | --- | --- | --- | --- | --- | --- | --- |
| Intercept1 | | -1.6797 | 0.9004 | | 3.4797 | | 0.0621 | |  | |  |
| Intercept2 | | -0.9625 | 0.8413 | | 1.3087 | | 0.2526 | |  | |  |
| INR | | 0.2733 | 0.1347 | | 4.1177 | | 0.0424 | | 1.314 | | 1.009-1.711 |
