# Supplementary material for: Infantile Hepatitis B in Immunized Children: Risk for Fulminant Hepatitis and Long-Term Outcomes
Source: PLoS One. 2014 Nov 7;9(11):e111825. doi: 10.1371/journal.pone.0111825 (PMC4224399; doi:10.1371/journal.pone.0111825)
Supplement: Table S3 — Clinical characteristics and outcome of 20 infants with non-fulminant hepatitis B. (DOCX) [file pone.0111825.s003.docx]

Table S3. Clinical characteristics and outcome of 20 infants with non-fulminant hepatitis B

|  | Recovery  (n=13) | Chronic carrier  (n=7) | *P*-value |
| --- | --- | --- | --- |
| **Demographic data** |  |  |  |
| Sex (M/F) | 10/3 | 5/2 | 1.0 |
| Age of onset (m) | 3.39 (2.01-11.64) | 7.69 (4.83-10.98) | 0.02 |
|  |  |  |  |
| **Maternal HBV status** |  |  |  |
| Maternal HBsAg (+) | 9/11 (82%) | 7/7 (100%) | 0.497 |
| Maternal HBeAg (+) | 4/11 (36%) | 7/7 (100%) | 0.01 |
|  |  |  |  |
| **Clinical data** |  |  |  |
| HBeAg (+) at enrollment | 3/10 (30%) | 7/7 (100%) | 0.01 |
| Initial ALT level (IU/L) | 872 (561-3020) | 427 (79-727) | <0.001 |
| Initial total bilirubin (mg/dl) | 5.7 (0.7-11.5) | 0.4 (0.2-6.6) | 0.02 |
| Initial INR | 1.5 (0.9-1.7) | 1.0 (0.9-1.5) | 0.02 |
| Peak ALT level (IU/L) | 925 (561-3020) | 514(129-1430) | 0.006 |
| Peak total bilirubin (mg/dl) | 5.8 (2.2-14.6) | 1.2 (0.4-13.1) | 0.02 |

HBsAg, hepatitis B surface antigen; HBeAg, hepatitis B e antigen, HBV, hepatitis B virus;

HBIG, hepatitis B immunoglobulin; ALT, alanine aminotransferase; INR, international normalized ratio
